# Determinants of Reduced Antiplatelet Effect of Aspirin in Patients with Stable Coronary Artery Disease

**DOI:** 10.1371/journal.pone.0126767

**Published:** 2015-05-18

**Authors:** Sanne Bøjet Larsen, Erik Lerkevang Grove, Søs Neergaard-Petersen, Morten Würtz, Anne-Mette Hvas, Steen Dalby Kristensen

**Affiliations:** 1 Department of Cardiology, Aarhus University Hospital, Aarhus, Denmark; 2 Department of Clinical Biochemistry, Aarhus University Hospital, Aarhus, Denmark; 3 Faculty of Health Sciences, Aarhus University, Aarhus, Denmark; Medizinische Hochschule Hannover, GERMANY

## Abstract

**Background:**

Aspirin is a cornerstone in management of coronary artery disease (CAD). However, considerable variability in the antiplatelet effect of aspirin has been reported.

**Aim:**

To investigate independent determinants of reduced antiplatelet effect of aspirin in stable CAD patients.

**Methods:**

We performed a cross-sectional study including 900 stable, high-risk CAD patients. Among these, 795 (88%) had prior myocardial infarction, 250 (28%) had type 2 diabetes, and 170 (19%) had both. All patients received 75 mg aspirin daily as mono antiplatelet therapy. The antiplatelet effect of aspirin was assessed by measurement of platelet aggregation employing 1) multiple electrode aggregometry (MEA, Multiplate Analyzer) in whole blood anticoagulated with citrate or hirudin using arachidonic acid (AA) or collagen as agonists, and 2) VerifyNow Aspirin Assay. Compliance was assessed by measurement of serum thromboxane B_2_.

**Results:**

Platelet count, prior myocardial infarction, type 2 diabetes and body mass index were independent determinants of increased AA-induced MEA platelet aggregation in citrate and hirudin anticoagulated blood (p-values ≤ 0.045). Similar results were found with VerifyNow. Prior coronary artery bypass grafting, age, smoking (MEA, AA/citrate) and female gender (MEA, AA/hirudin) were also independent determinants of increased platelet aggregation (p-values ≤ 0.038). Compliance was confirmed by low serum thromboxane B_2_ levels in all patients (median [25%;75%]: 0.97 [0.52;1.97], range 0.02-26.44 ng/ml).

**Conclusion:**

Platelet count, prior myocardial infarction, type 2 diabetes and body mass index were independent determinants of increased platelet aggregation, indicating that these characteristics may be key factors in reduced antiplatelet effect of aspirin in stable CAD patients.

## Introduction

Low-dose aspirin is recommended for patients with stable coronary artery disease (CAD) [1]. Regardless of the well-known beneficial antiplatelet effect of aspirin, a substantial proportion of patients with CAD display considerable variability in the effect of aspirin [2]. Two meta-analyses have reported that reduced antiplatelet effect of aspirin entails a nearly four-fold increased risk of adverse cardiovascular events [3,4]. Despite extensive research on variability in the antiplatelet effect of aspirin, several issues contribute to the difficulty and complexity of data interpretation. These issues include inconsistent definitions of “aspirin resistance”, differences in platelet function tests including agonists and anticoagulants used as well as cut-off levels applied to define the prevalence of "low-responders", and small or heterogeneous study populations [2,5]. Finally, in many previous studies, compliance has not been evaluated properly.

Most likely, variability in the antiplatelet effect of aspirin is multifactorial and include genetic, biological and clinical factors [2,6]. Previous studies have suggested that some patients are more likely to have reduced antiplatelet effect of aspirin [7]. Thus, we hypothesised that prior myocardial infarction (MI), prior coronary artery bypass grafting, (CABG), prior stroke, type 2 diabetes mellitus, age, female gender, obesity, current smoking, renal insufficiency and platelet count were potential modifiers of the antiplatelet effect of aspirin.

The aim of the study was to investigate independent determinants of reduced antiplatelet effect of aspirin in a large cohort of stable CAD patients using two platelet aggregation tests, two agonists and two anticoagulants.

## Materials and Methods

### Study population and design

The study was a cross-sectional study including 900 patients with stable CAD. Data for the present study is based on data from four previous studies by our group [8–11]. From November 2007 to January 2011, patients were recruited from the Western Denmark Heart Registry, which collects data on all interventional procedures performed in the Western part of Denmark [12].

The study cohort represents a high-risk CAD population since all patients had angiographically documented CAD and either prior MI, type 2 diabetes mellitus or both. Patients with recent cardiovascular events were excluded in order to avoid dual antiplatelet therapy. Concordance with inclusion and exclusion criteria was checked using telephone interviews, medical records and blood samples. The inclusion criteria were: a) age ≥ 18 years, b) daily treatment with aspirin, c) significant CAD verified by prior percutaneous coronary intervention (PCI), CABG, or by a coronary angiography showing at least one 50% coronary luminal narrowing, d) patients with prior MI at least 12 months ago verified by electrocardiographic ST-segment elevation and/or elevated plasma troponin T (> 0.10 μg/l) and/or plasma creatine kinase-MB (> 12 U/l). The exclusion criteria were: a) ongoing medical treatment known to affect platelet function or coagulation (e.g. non-steroidal anti-inflammatory drugs, any anticoagulants or antiplatelet drugs except aspirin), b) any ischaemic vascular event, PCI, or CABG within the previous 12 months, c) platelet count < 120 x 10^9^/l or > 450 x 10^9^/l, d) inability to give informed consent. All diabetic patients were diagnosed with type 2 diabetes and treated with oral antidiabetic drugs and/or insulin. All non-diabetic patients had fasting plasma glucose levels < 7.0 mmol/l at the time of inclusion.

The study was conducted in agreement with the Helsinki-II-declaration and approved by The Central Denmark Region Committees on Health Research (project # 2007–0180, 2008–0188, 2008–0189, M-2009-0110) and by the Danish Data Protection Agency. All patients gave written informed consent.

### Compliance

To optimise compliance and uniform pharmacokinetics, all patients received a pill box with seven tablets of 75 mg non-enteric coated aspirin (Hjerdyl, Sandoz, Denmark). This ensured that all patients included in the study received the exact same aspirin dose and preparation prior to and at the time of blood sampling. On the day of blood sampling, patients were instructed to ingest aspirin exactly one hour before blood sampling. Compliance with aspirin was optimised by confirmation of empty pill boxes and questioning about aspirin intake. Finally, measurement of serum thromboxane B_2_ (TXB_2_) was performed in all patients.

### Laboratory investigations

#### Blood sampling

Standardised blood sampling was performed one hour after aspirin ingestion. Blood samples were obtained from the antecubital vein with patients in supine position after 30 minutes of rest using vacuum tubes, a large bore needle (19 G), and a minimum of stasis.

#### Haematology

Blood samples for haematological analyses were collected in 3.0 ml tubes containing EDTA (Terumo, Leuven, Belgium) and analysed within 90 minutes of sampling. Haematology parameters were measured employing a Sysmex XE-2100 haematology analyser (Sysmex, Kobe, Japan).

#### Platelet aggregation tests

Platelet aggregation was evaluated with two different instruments using whole blood: multiple electrode aggregometry (MEA, Multiplate Analyzer, Roche Diagnostics International LDT, Rotkreuz, Switzerland) and VerifyNow Aspirin Assay (Accumetrics Inc., San Diego, CA, USA).

For MEA, 3.6 ml tubes containing 3.2% sodium citrate (Terumo, Leuven, Belgium) or 3.0 ml tubes containing hirudin at a final concentration of 25 μg/ml (Terumo, Leuven, Belgium) were utilised. MEA platelet aggregation was induced using arachidonic acid (AA) 1.0 mM (ASPI test, Triolab AS, Brøndby, Denmark) or collagen 1.0 μg/ml (Horm, Medinor, Nycomed, Austria). Blood samples rested for at least 30 minutes before analysis but no longer than 120 minutes. Aggregation was reported as the area under the curve (aggregation units [AU]*min). In case of a deviation > 20% between the two impedance curves, the sample was re-analysed. The reproducibility of these platelet aggregation tests has previously been reported by our group [13].

For VerifyNow analyses, blood was collected in 2.7 ml tubes containing 3.2% sodium citrate (Terumo, Leuven, Belgium). The VerifyNow system uses disposable cartridges with four mixing chambers containing fibrinogen-coated beads and AA as agonist. Blood samples rested for at least 30 minutes before analysis but no longer than 120 minutes. Platelet aggregation was reported as Aspirin Reaction Units (ARU).

#### Serum thromboxane B_2_.

Since aspirin specifically blocks the cyclooxygenase-1 enzyme mediating the conversion of AA to thromboxane, measurement of this metabolite is considered the most pharmacologically specific analysis of platelet inhibition with aspirin [14]. We measured serum TXB_2_ using enzyme-linked immunosorbent assay (ELISA) according to the manufacturer’s instructions (Thromboxane B_2_ EIA Kit, Cayman Chemical, MI, USA). Blood was collected in non-siliconised tubes without anticoagulant and allowed to clot for one hour at 37°C. Subsequently, samples were centrifuged for 10 minutes at 2600 *g*, and serum was removed and stored at -80°C until analysis.

### Statistics

Continuous data are presented as mean and standard deviation (SD) if normally distributed and if not, as median and interquartile range. Differences between two unpaired groups were tested with a two-sided t-test if data were normally distributed and if not, the Mann-Whitney test was used. Differences between two paired groups were tested using a paired t-test if data were normally distributed and if not, the Wilcoxon signed rank test was used. Proportions between two groups were tested using Fisher’s exact test and presented as absolute counts and percentages. Correlations were calculated using Spearman’s rank coefficient. Multiple linear regression analyses were used to identify independent determinants of platelet aggregation. In the regression analyses, observations were removed if they were missing on the outcome variable or on any of the predictor variables. A two-sided p-value < 0.05 was considered statistically significant. To avoid typing errors, data were registered in Epidata version 3.1 (EpiData Association, Denmark). Statistical analyses were performed using Stataversion 11.0 (StataCorp, College Station, TX, USA) and graphs were made using GraphPad Prism version 5.0 (GraphPad Software, San Diego, CA, USA).

## Results

### Study population

Clinical and biochemical characteristics of the study population are shown in Table 1. We studied a population of stable CAD patients with a relatively high-risk profile, since 795 (88%) of the patients had a history of MI, 250 (28%) had type 2 diabetes and 170 (19%) had both. Compliance was confirmed by low serum TXB_2_ levels in all patients (median [25%;75%]: 0.97 [0.52;1.97], range 0.02–26.44 ng/ml) corresponding to ≥95% inhibition of platelet cyclooxygenase-1 activity [15].

**Table 1:** Baseline characteristics of the study population, n = 900.

Age, years	65±4
Body mass index, kg/m^2^	27±8
Males	704(78)
Current smokers	199(22)
Blood pressure, systolic, mm Hg	142±20
Blood pressure, diastolic, mm Hg	83±12
**Biochemistry**	
B-Leukocyte count, 10^9^/l^a^	7.1±1.9
B-Haemoglobin, mmol/l	8.8±0.8
B-Red blood cell count, 10^12^/l^b^	4.7±0.4
B-Platelet count, 10^9^/l	233±58
P-Creatinine, μmol/l	82(71;96)
B-estimated glomerular filtration rate, ml/min	78(65;90)
**Morbidity**	
Prior percutaneus coronary intervention	849(94)
Prior myocardial infarction	795(88)
Prior coronary artery bypass grafting	122(14)
Prior stroke	53(6)
Type 2 diabetes mellitus	250(28)
**Medication**	
Aspirin	900(100)
Statins	813(90)
Beta-blockers	682(76)
ACE inhibitors	424(47)
Angiotensin receptor blockers	139(15)
Calcium antagonists	194(22)
Diuretics	272(30)
Proton pump inhibitors	105(12)
Insulin^c^	76(30)
Oral antidiabetic medication^c^	205(82)

Data is presented as mean±SD, n(%) or median (25th;75th percentile)

^a^ Leucocyte count only available for 714 patients

^b^ Red blood cell count only available for 751 patients

^c^ out of 250 patients with type 2 diabetes

B: blood, S: serum, P: plasma, ACE: Angiotensin converting enzyme

### Platelet aggregation

**Fig 1:**
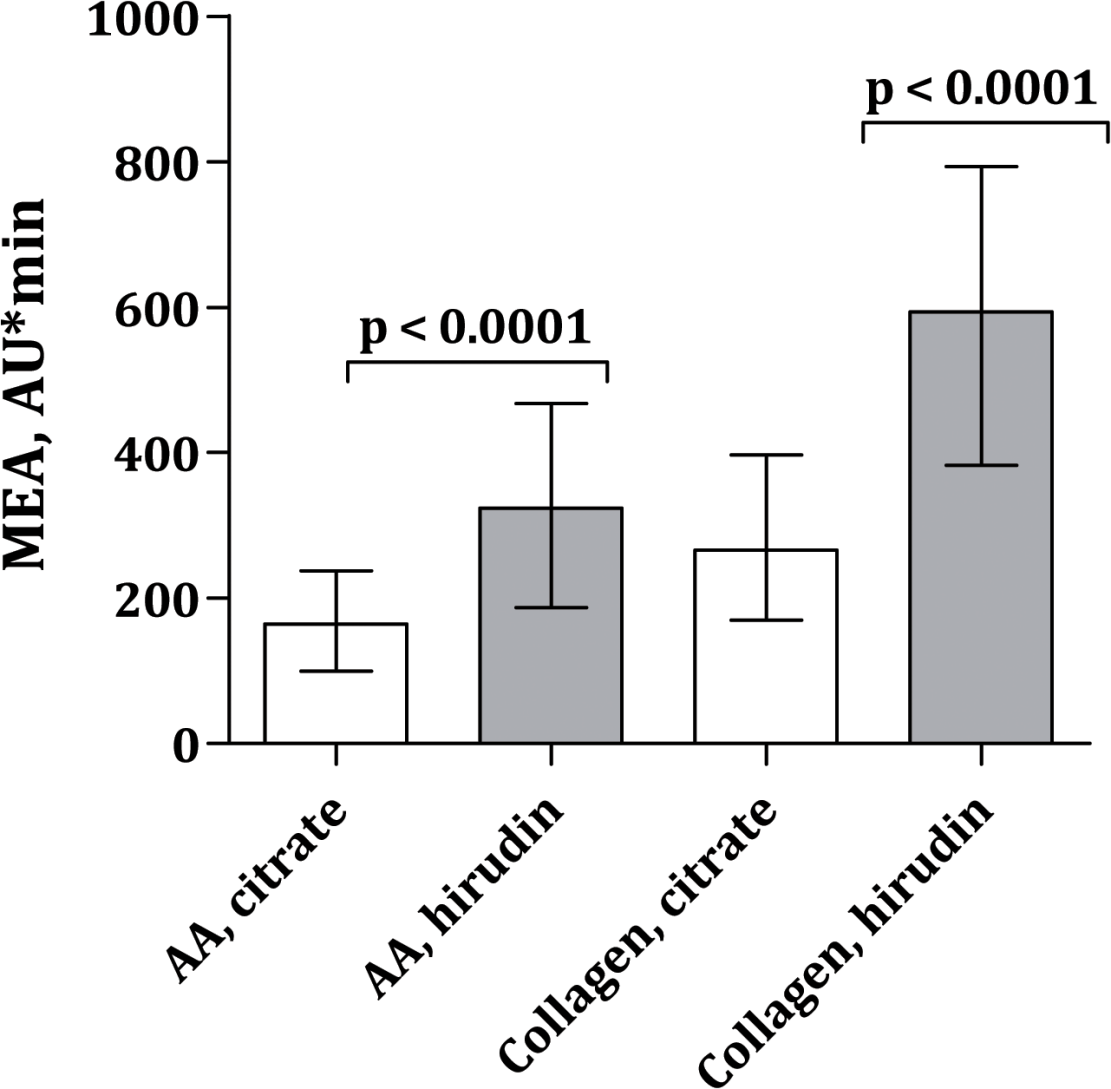
MEA platelet aggregation levels with arachidonic acid (AA) or collagen as agonist and citrate or hirudin as anticoagulant. Data is presented with median and interquartile range. Out of 900 patients, AA/citrate MEA platelet aggregation results were available for 892 patients, AA/hirudin results were available for 751 patients, collagen/citrate results were available for 895 patients and collagen/hirudin results were available for 752 patients.

MEA platelet aggregation levels were (median AU*min [25%;75%]) 165 (100;238) for AA/citrate, 324 (187;468) for AA/hirudin, 266 (170;397) for collagen/citrate and 594 (382;794) for collagen/hirudin (Fig 1). VerifyNow platelet aggregation levels were 426 (409;452) ARU. Age ≥ 65 years, female sex, body mass index ≥ 30 kg/m^2^, type 2 diabetes and platelet count ≥ 226 x 10^9^/L were significantly associated with elevated platelet aggregation levels (Table 2).

**Table 2:** Platelet aggregation levels according to gender, age, body mass index, type 2 diabetes and platelet count, n = 900.

		Multiplate		Multiplate			
		AA, citrate		AA, hirudin			
	*n (%)*		*p-value*	* *	*p-value*		
***Gender***							
**Females**	196 (22)	191 (125;285)		325 (168;506)			
**Males**	704 (78)	157 (97;227)	p = 0.0008	324 (190;452)	p = 0.82		
***Age***							
**< 65years**	409 (45)	154 (90;233)		329 (190;472)			
**≥ 65 years**	491 (55)	169 (108;243)	p = 0.01	309 (185;464)	p = 0.33		
***Body mass index***							
**< 30 kg/m2**	658 (73)	158 (98;230)		309 (171;448)			
**≥ 30 kg/m2**	242 (27)	172 (114;269)	p = 0.02	359 (225;509)	p = 0.0004		
***Type 2 diabetes***							
**No**	650 (72)	156 (97;224)		307 (173;438)			
**Yes**	250 (28)	188 (112;282)	p = 0.0001	385 (232;519)	p < 0.0001		
***Platelet count*** *							
**< 226 x 10** ^**9**^ **/L**	445 (49)	134 (85;201)		256 (162;389)			
**≥ 226 x 10** ^**9**^ **/L**	455 (51)	192 (122;287)	p < 0.0001	382 (234;512)	p < 0.0001		
** **		**Multiplate**		**Multiplate**		**VerifyNow**	
** **		**collagen, citrate**		**collagen, hirudin**		**AA**	
** **	*n (%)*		*p-value*	* *	*p-value*	* *	*p-value*
***Gender***							
**Females**	196 (22)	314 (209;455)		650 (419;823)		424 (409;450)	
**Males**	704 (78)	255 (167;369)	p = 0.0002	583 (279;774)	p = 0.42	426 (409;454)	p = 0.48
***Age***							
**< 65years**	409 (45)	260 (155;396)		612 (420;789)		426 (408;453)	
**≥ 65 years**	491 (55)	271 (183;400)	p = 0.03	580 (355;799)	p = 0.63	425 (409;452)	p = 0.81
***Body mass index***							
**< 30 kg/m2**	658 (73)	264 (170;383)		583 (376;789)		423 (408;449)	
**≥ 30 kg/m2**	242 (27)	290 (173;428)	p = 0.24	630 (435;805)	p = 0.59	432 (412;464)	p = 0.0007
***Type 2 diabetes***							
**No**	650 (72)	262 (171;372)		570 (365;772)		423 (408;447)	
**Yes**	250 (28)	282 (170;438)	p = 0.01	662 (476;834)	p = 0.0006	437 (414;472)	p < 0.0001
***Platelet count*** *							
**< 226 x 10** ^**9**^ **/L**	445 (49)	228 (143;315)		516 (334;703)		423 (408;447)	
**≥ 226 x 10** ^**9**^ **/L**	455 (51)	329 (214;457)	p < 0.0001	685 (460;845)	p < 0.0001	429 (410;461)	p = 0.0006

Data is presented as median (25th;75th percentile).

*226 x 109/L was the median; accoring to the exclusion criteria, patients with platelet count < 120 x 109/L or > 450 x 109/L were excluded.

**Fig 2:**
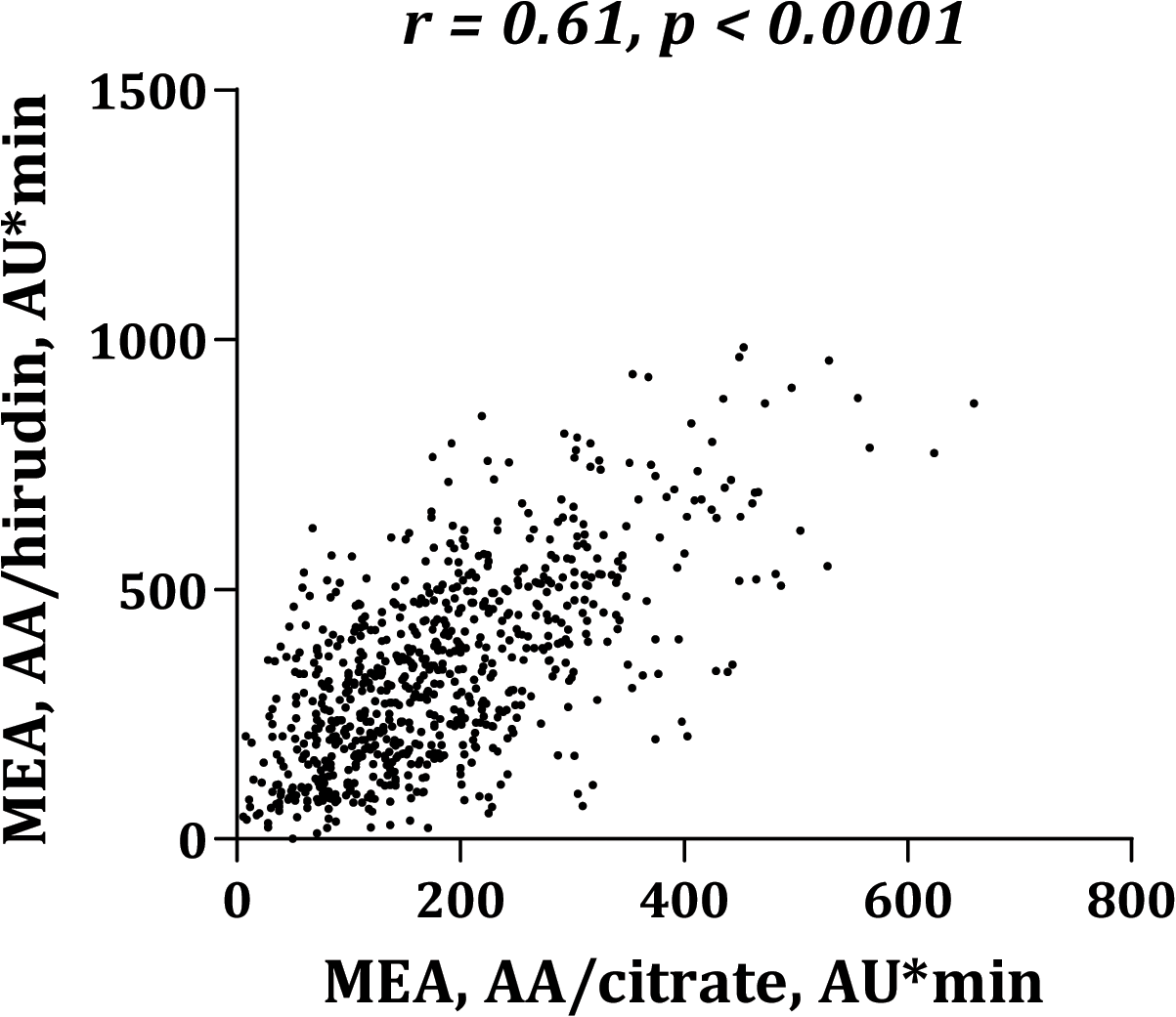
Correlation between multiple electrode aggregometry (MEA) with arachidonic acid (AA) as agonist and citrate or hirudin as anticoagulant.

**Fig 3:**
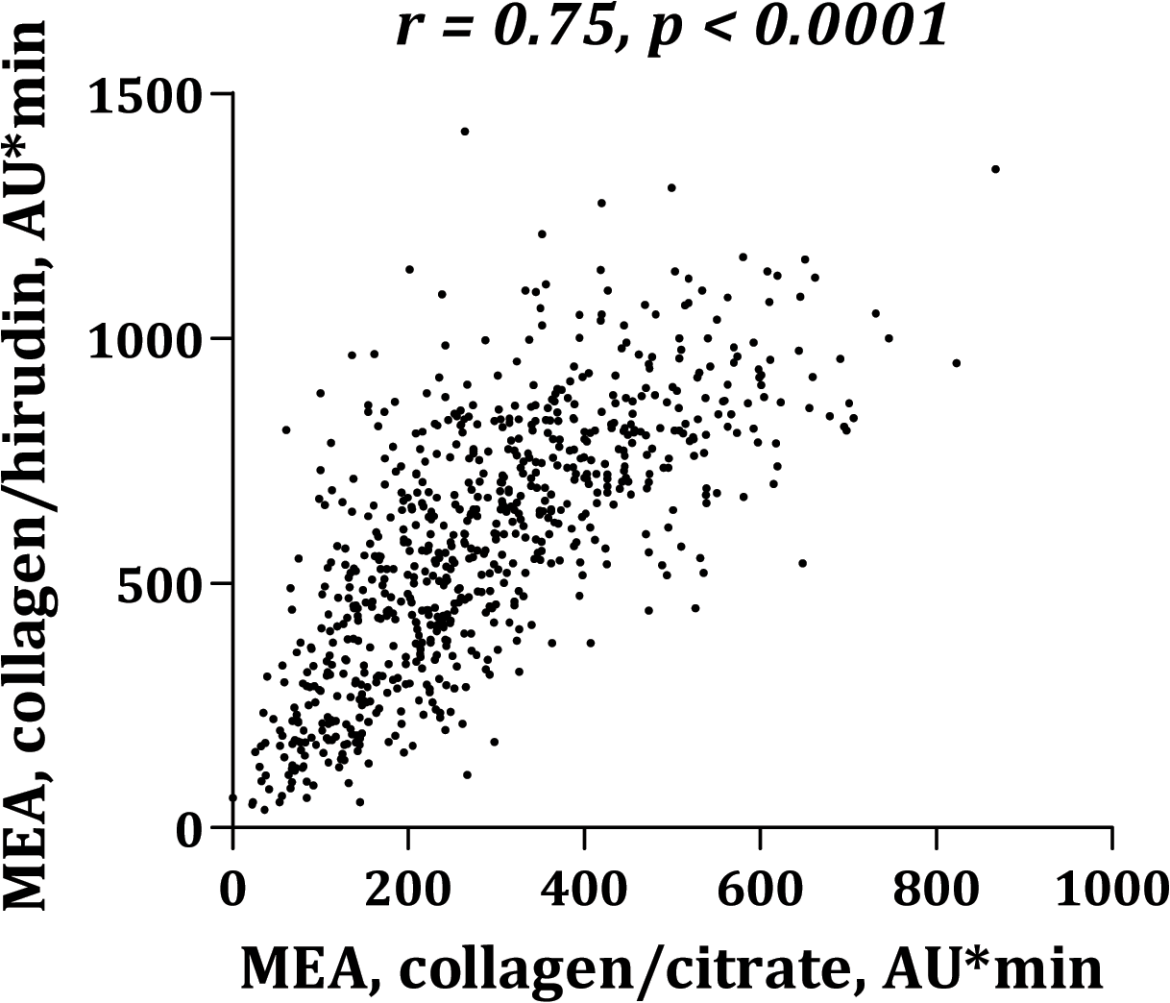
Correlation between multiple electrode aggregometry (MEA) with collagen as agonist and citrate or hirudin as anticoagulant.

**Fig 4:**
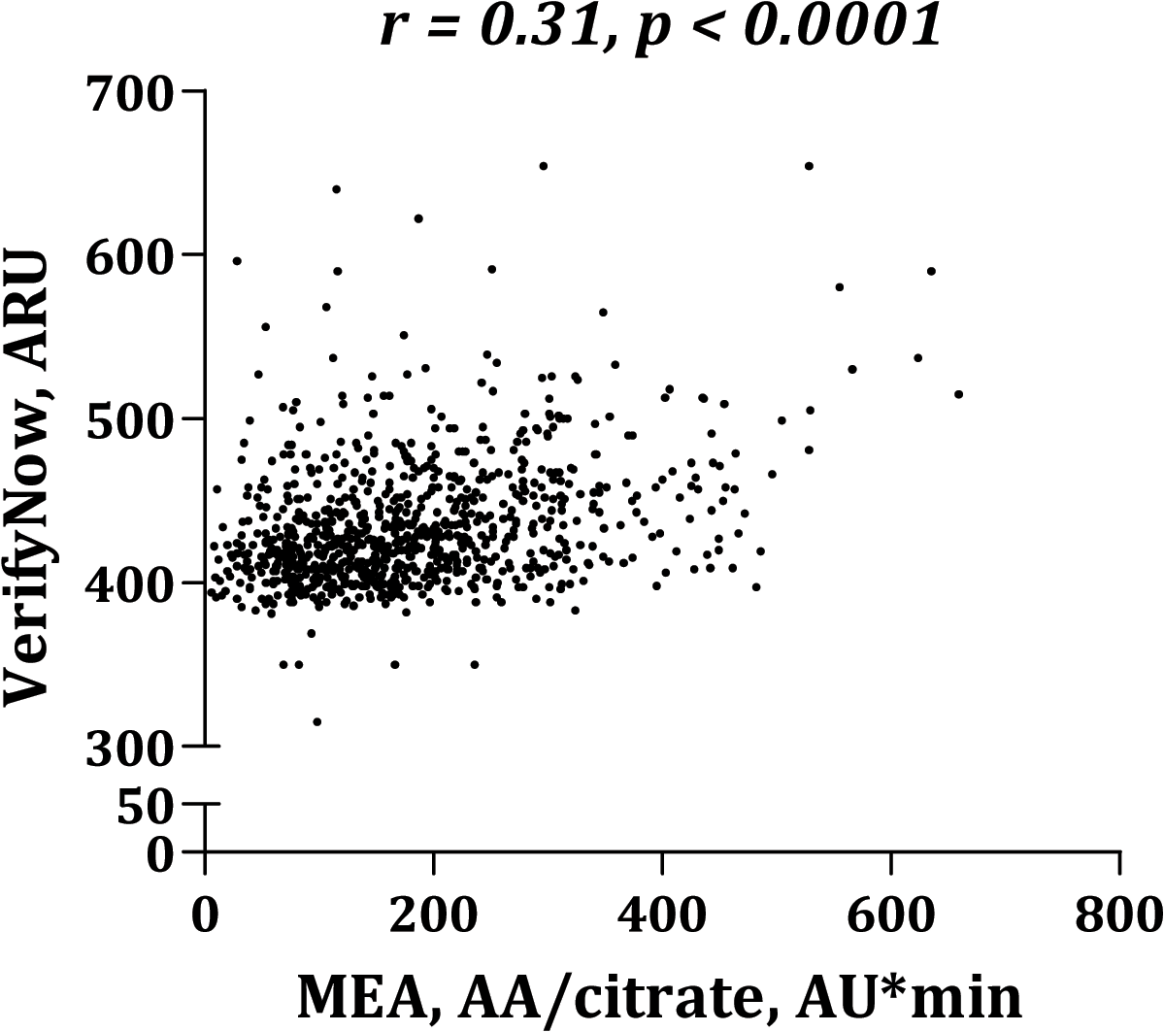
Correlation between VerifyNow and multiple electrode aggregometry (MEA) using arachidonic acid (AA) as agonist and citrate as anticoagulant.

**Fig 5:**
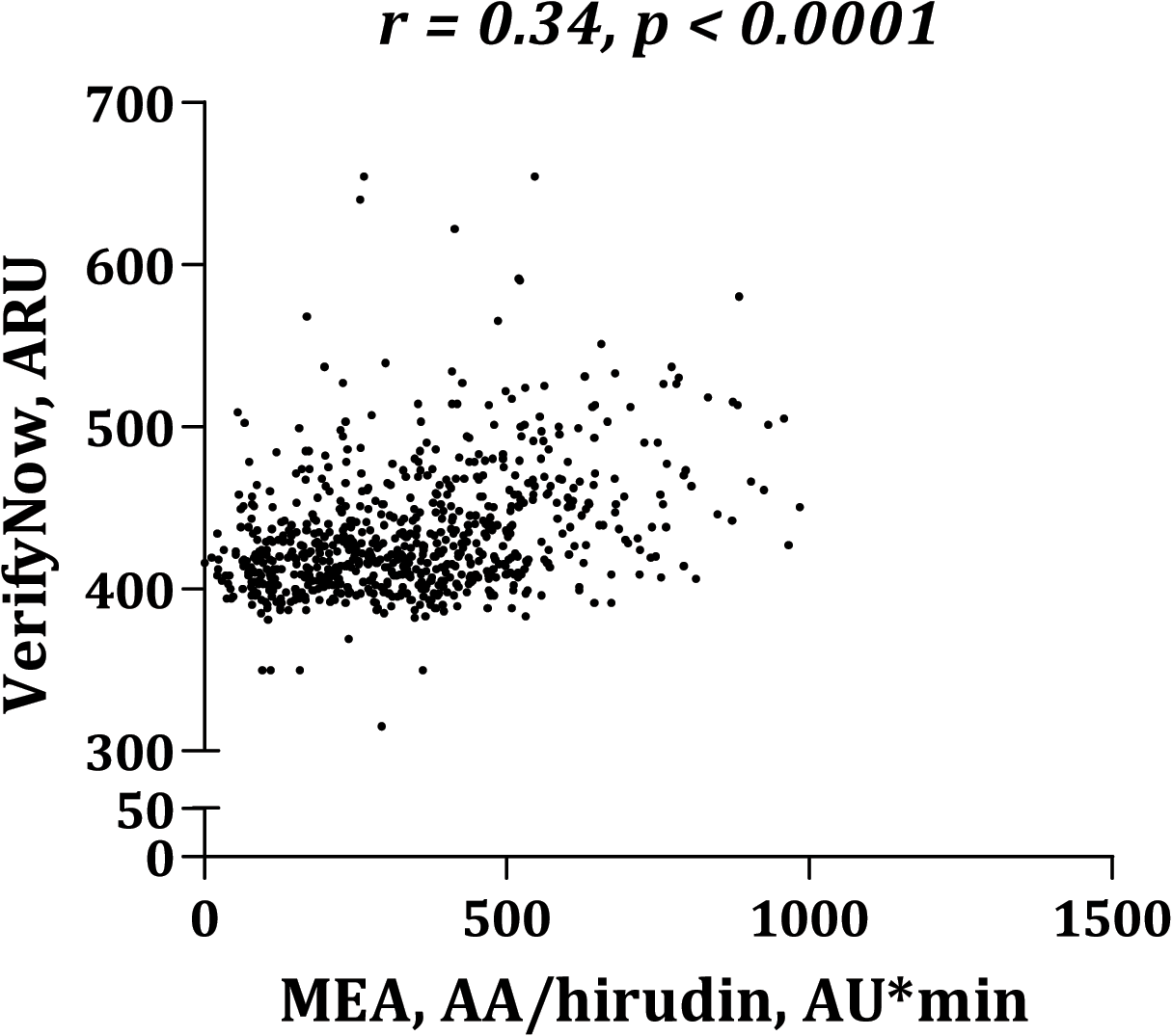
Correlation between VerifyNow and multiple electrode aggregometry (MEA) using arachidonic acid (AA) as agonist and hirudin as anticoagulant.

Platelet aggregation levels were significantly higher in blood anticoagulated with hirudin than with citrate (Fig 1). MEA platelet aggregation induced by AA (Fig 2) or collagen (Fig 3) correlated significantly whether using citrate or hirudin as anticoagulant. Similarly, there was a significant correlation between MEA and VerifyNow platelet aggregation (Figs 4 and 5).

### Determinants of reduced antiplatelet effect of aspirin

In order to identify independent determinants of reduced antiplatelet effect of aspirin, we performed multivariate linear regression analyses with platelet aggregation as dependent variables and prior MI, prior CABG, prior stroke, type 2 diabetes, age, female gender, body mass index, current smoking, renal insufficiency (estimated glomerular filtration rate [eGFR]) and platelet count as independent variables (Table 3).

**Table 3:** Independent determinants of platelet aggregation in stable CAD patients, n = 900.

	**Multiplate**		**Multiplate**			
	**AA, citrate**		**AA, hirudin**			
	Beta (95% CI)	p-value	Beta (95% CI)	p-value		
**Prior MI**	1.16 (1.00:1.35)	0.045	1.21 (1.01;1.44)	0.038		
**Prior CABG**	1.17 (1.03;1.33)	0.014	1.08 (0.93;1.26)	0.326		
**Prior stroke**	1.07 (0.89,1.28)	0.487	0.96 (0.77;1.19)	0.687		
**Type 2 diabetes**	1.16 (1.03;1.29)	0.010	1.28 (1.12;1.46)	<0.0001		
**Age, years**	1.01 (1.00;1.01)	0.004	1.00 (0.99;1.00)	0.262		
**Female sex**	1.06 (0.95;1.18)	0.325	0.88 (0.78;0.99)	0.035		
**Current smoking**	1.13 (1.01;1.25)	0.026	1.09 (0.97;1.22)	0.168		
**Body mass index, kg/m** ^**2**^	1.02 (1.01;1.03)	0.004	1.01 (1.00;1.03)	0.016		
**eGFR, mL/min**	1.00 (1.00;1.00)	0.314	1.00 (0.99;1.00)	0.245		
**Platelet count, 10** ^**9**^ **/L**	1.00 (1.00;1.00)	<0.0001	1.00 (1.00;1.00)	<0.0001		
	**Multiplate**		**Multiplate**		**VerifyNow**	
	**collagen, citrate**		**collagen, hirudin**		**AA**	
	Beta (95% CI)	p-value	Beta (95% CI)	p-value	Beta (95% CI)	p-value
**Prior MI**	1.06 (0.93;1.21)	0.396	72.01 (3.69;140.32)	0.039	0.99 (0.97;1.01)	0.375
**Prior CABG**	0.97 (0.87;1.09)	0.648	-34.78 (-94:19;24.61)	0.251	1.00 (0.98;1.91)	0.891
**Prior stroke**	1.06 (0.90;1.25)	0.505	-27.74 (-111.69;56.22)	0.517	1.01 (0.98;1.03)	0.557
**Type 2 diabetes**	1.08 (0.98;1.20)	0.113	85.34 (34.86;135.81)	0.0001	1.02 (1.01;1.03)	0.004
**Age, years**	1.01 (1.00;1.01)	<0.0001	-0.15 (-2.37;2.08)	0.897	1.00 (1.00;1.00)	0.181
**Female sex**	1.07 (0.97;1.18)	0.189	-6.16 (-51.31;40.98)	0.826	0.99 (0.97;1.00)	0.070
**Current smoking**	0.96 (0.88;1.06)	0.431	4.76 (-40.63;50.16)	0.837	1.01 (0.99;1.02)	0.246
**Body mass index, kg/m** ^**2**^	1.00 (0.99;1.01)	0.426	0.97 (-3.59;5.53)	0.677	1.00 (1.00;1.00)	<0.0001
**eGFR, mL/min**	1.00 (1.00;1.01)	0.026	0.64 (-0.59;1.88)	0.305	1.00 (1.00;1.00)	0.267
**Platelet count, 10** ^**9**^ **/L**	1.00 (1.00;1.00)	<0.0001	1.16 (0.82;1.49)	<0.0001	1.00 (1.00;1.00)	<0.0001

AA: arachidonic acid, CI: confidence interval, MI: myocardial infarction, CABG: coronary artery bypass grafting, eGFR: estimated glomerular filtration rate

High platelet count was an independent determinant of increased platelet aggregation assessed by both platelet aggregation tests, both agonists and both anticoagulants (all p-values < 0.0001) (Table 3). Additionally, prior MI, type 2 diabetes and high body mass index independently predicted increased AA-induced MEA platelet aggregation whether using citrate or hirudin as anticoagulant (p-values ≤ 0.045). Type 2 diabetes and body mass index were also determinants of VerifyNow platelet aggregation (p ≤ 0.004). Furthermore, prior coronary artery bypass grafting, age and smoking independently predicted AA/citrate MEA platelet aggregation (p-values ≤ 0.026) and female gender independently predicted AA/hirudin MEA platelet aggregation (p = 0.035). Age and eGFR predicted collagen/citrate MEA platelet aggregation (p-values ≤ 0.026), whereas prior MI and type 2 diabetes predicted collagen/hirudin MEA platelet aggregation (p-values ≤ 0.039).

AA-induced MEA platelet aggregation levels were increased by 16–21% in patients with prior MI compared with patients without prior MI (p-values ≤ 0.045) and by 16–28% in patients with type 2 diabetes compared with patients without diabetes (p-values ≤ 0.010) whether using citrate or hirudin as anticoagulant. When patients with prior MI and type 2 diabetes were pooled in one group and compared with patients without prior MI and type 2 diabetes, no additional increase in AA-induced MEA aggregation was observed (data not shown, 18–23%, p-values ≤ 0.004).

## Discussion

This is the largest study so far to investigate independent determinants of reduced antiplatelet effect of aspirin in stable, high-risk CAD patients receiving aspirin as mono antiplatelet therapy. We found that platelet count, prior MI, type 2 diabetes and body mass index were independent determinants of a reduced antiplatelet effect of aspirin.

### Determinants of reduced antiplatelet effect of aspirin

In the present study, high platelet count was an independent determinant of increased platelet aggregation with MEA as well as with VerifyNow. These results concur with previous findings from our group suggesting that MEA whole blood aggregometry is dependent on platelet count in stable CAD patients [16]. Extending these results, the present study showed that platelet aggregation evaluated by VerifyNow is also dependent on platelet count, even within the normal range of platelet count. In studies performing platelet aggregation analyses using light transmission aggregometry [17] or the Platelet Function Analyzer-100 [18], an association between platelet count and platelet aggregation in stable CAD patients has also been reported.

Prior MI was an independent determinant of MEA platelet aggregation, which is consistent with previous findings in smaller studies [8,19]. Compared with healthy individuals, CAD patients have circulating activated platelets, circulating monocyte-platelet aggregates and increased platelet reactivity, which may indicate a pro-thrombotic profile [20]. In CAD patients with prior MI, reduced antiplatelet effect of aspirin may be associated with more severe atherosclerosis [21]. Another hypothesis attributes to increased platelet turnover, which has attracted increasing attention as a mechanism explaining reduced antiplatelet drug efficacy in CAD patients [22,23]. One may hypothesise that patients with prior MI have increased platelet turnover, which could at least partly explain our results. Supporting this hypothesis, Hamsten et al. demonstrated shortened megakaryocyte-platelet regeneration in patients with prior MI [24].

Patients with type 2 diabetes are known to carry an increased risk of cardiovascular events, most likely explained by clustering of risk factors such as hypertension, dyslipidemia, overweight, impaired fibrinolysis, insulin resistance, hyperglycaemia as well as endothelial and platelet dysfunction [25]. Several of these factors profoundly influence platelet activation and aggregation, thereby inducing platelet hyper-reactivity and a pro-coagulant environment in diabetics [26]. In the present study, type 2 diabetes was an independent determinant of increased platelet aggregation evaluated by both MEA and VerifyNow. These findings extend results from previous smaller studies evaluating the effect of aspirin in patients with diabetes [25,27,28].

We found that high body mass index was an independent determinant of increased AA-induced platelet aggregation using MEA and VerifyNow, which is consistent with results found in healthy individuals [29] as well as in smaller studies including CAD patients [19,30]. Obesity affects pharmacokinetics on the basis of changes in distribution volume, regional blood flow and renal and hepatic clearance mechanisms [21]. Growing evidence suggests that obesity, independent of its associated comorbid conditions, may be associated with reduced antiplatelet drug efficacy [31,32]. A faster bioinactivation of aspirin related to modifications of pharmacokinetic mechanisms associated with obesity may partially explain this finding [33]. Also leptin, a hormone that is elevated in obesity, has been suggested to reduce aspirin efficacy through prothrombotic effects [34].

Age, female gender, current smoking, prior CABG and eGFR were also independent determinants of platelet aggregation, however, these results were not consistent across assays, agonists and anticoagulants. In our study, increasing age predicted augmented platelet aggregation independent of traditional risk factors such as gender, prior MI, diabetes, smoking and body mass index. Thus, our results extend previous findings in CAD patients receiving single [35] or dual antiplatelet therapy [36].

Gender was an independent predictor of AA-induced MEA platelet aggregation with an increased antiplatelet effect of aspirin in women. This finding extends previous studies showing that low-dose aspirin produces greater platelet inhibition in healthy women than in healthy men when evaluated with AA as agonist. Yet, when evaluating platelet aggregation induced by collagen, women had lower antiplatelet effect of aspirin than men (data not shown), which is in accordance with previous studies [37,38]. Thus, reduced antiplatelet effect of aspirin in women may be mediated through COX-1-independent pathways.

Smoking independently predicted increased AA-induced MEA platelet aggregation, mirroring previous results based on smaller and more heterogeneous study populations employing different platelet function tests [39,40].

### Anticoagulants

Measurement of platelet aggregation is dependent on a number of factors including the assay employed, cut-off limits, agonists, storage time, temperature and anticoagulants [41]. Thus, the heterogeneity of these factors in previous studies may partially explain the differing variability in the antiplatelet effect of aspirin. In our study, all pre-analytical procedures and platelet aggregation analyses were strictly standardised. We found consistently higher MEA platelet aggregation levels in hirudinised blood than in citrated blood, which is in line with previous studies [42,43]. Our results most likely reflect the importance of extracellular calcium for platelet aggregation. Importantly, the lower platelet aggregation levels in citrated samples could lead to overestimation of antiplatelet drug efficacy, which should be kept in mind when interpreting platelet aggregation results. The direct thrombin inhibitor hirudin maintains physiological levels of divalent cations and therefore, analysing platelet aggregation with hirudin may be preferred [44].

### Strengths and limitations

The overall strength of this study is the inclusion of a large study population with stable CAD. Although we studied high-risk CAD patients, our study population is similar to a large fraction of everyday CAD patients. Thus, the study results have high external validity. Pre-analytical procedures, aspirin dosing and the interval from aspirin intake to platelet aggregation analysis were strictly standardised. Furthermore, compliance with aspirin was confirmed in all patients by measurement of serum TXB_2_.

In this observational study, we were not able to establish causality between patient characteristics and platelet aggregation. Further limitations include that no off-treatment baseline measurements were performed because discontinuation of aspirin therapy for study purposes was considered unethical in this high-risk CAD population.

### Conclusion

In the present study, platelet count, prior MI, type 2 diabetes and body mass index were independent determinants of increased platelet aggregation during low-dose aspirin treatment as evaluated with both MEA and VerifyNow. Prior CABG, age, female gender, current smoking and eGFR also predicted platelet aggregation levels, however, these results were not consistent across assays, agonists and anticoagulants. Our results indicate that a number of clinical characteristics are associated with reduced antiplatelet effect of aspirin in stable CAD patients.
